# The Influence of Betulin and Its Derivatives EB5 and ECH147 on the Antioxidant Status of Human Renal Proximal Tubule Epithelial Cells

**DOI:** 10.3390/ijms23052524

**Published:** 2022-02-25

**Authors:** Celina Kruszniewska-Rajs, Barbara Strzałka-Mrozik, Magdalena Kimsa-Dudek, Agnieszka Synowiec-Wojtarowicz, Elwira Chrobak, Ewa Bębenek, Stanisław Boryczka, Stanisław Głuszek, Joanna Magdalena Gola

**Affiliations:** 1 Department of Molecular Biology, Faculty of Pharmaceutical Sciences in Sosnowiec, Medical University of Silesia, 40-055 Katowice, Poland; ckruszniewska@sum.edu.pl (C.K.-R.); jgola@sum.edu.pl (J.M.G.); 2 Department of Nutrigenomics and Bromatology, Faculty of Pharmaceutical Sciences in Sosnowiec, Medical University of Silesia, 40-055 Katowice, Poland; mkimsa@sum.edu.pl (M.K.-D.); asynowiec@sum.edu.pl (A.S.-W.); 3 Department of Organic Chemistry, Faculty of Pharmaceutical Sciences in Sosnowiec, Medical University of Silesia, 40-055 Katowice, Poland; echrobak@sum.edu.pl (E.C.); ebebenek@sum.edu.pl (E.B.); boryczka@sum.edu.pl (S.B.); 4 Department of Surgical Medicine with the Laboratory of Medical Genetics, Institute of Medical Sciences, Collegium Medicum, Jan Kochanowski University, 25-317 Kielce, Poland; sgluszek@wp.pl; 5 Department of Clinic General Oncological and Endocrinological Surgery, Regional Hospital, 25-317 Kielce, Poland

**Keywords:** betulin, betulin derivatives, oxidative stress, renal proximal tubule epithelial cells

## Abstract

Betulin and its derivatives, 28-propyne derivative EB5 and 29-diethyl phosphonate analog ECH147, are promising compounds in anti-tumor activity studies. However, their effect on kidney cells has not yet been studied. The study aimed to determine whether betulin and its derivatives—EB5 and ECH147—influence the viability and oxidative status of human renal proximal tubule epithelial cells (RPTECs). The total antioxidant capacity of cells (TEAC), lipid peroxidation product malondialdehyde (MDA) level, and activity of antioxidant enzymes (SOD, CAT, and GPX) were evaluated. Additionally, the mRNA level of genes encoding antioxidant enzymes was assessed. Cisplatin and 5-fluorouracil were used as reference substances. Betulin and its derivatives affected the viability and antioxidant systems of RPTECs. Betulin strongly reduced TEAC in a concentration-dependent manner. All tested compounds caused an increase in MDA levels. The activity of SOD, CAT, and GPX, and the mRNA profiles of genes encoding antioxidant enzymes depended on the tested compound and its concentration. Betulin showed an cisplatin-like effect, indicating its nephrotoxic potential. Betulin derivatives EB5 and ECH147 showed different impacts on the antioxidant system, which gives hope that these compounds will not cause severe consequences for the kidneys in vivo.

## 1. Introduction

The richest source of betulin, the lupane-type triterpenoid (lup-20 (29)-en-3β,28-diol), is the outer bark of the trees of the genus *Betula* [1]. Numerous studies of betulin have shown that it has anti-inflammatory, antiviral, antibacterial, hepatoprotective, antioxidant, and anti-ulcer effects. However, researchers are particularly interested in its anticancer properties [2,3].

The introduction of various substituents into betulin 1 at position C-28 may favorably influence its pharmacological properties. The attachment of compounds containing a fragment with a carbon–carbon triple bond seems to be promising in the context of anti-tumor activity. This allows not only for the potential enhancement of the anticancer effect but also allows for further chemical modifications of the derivatives obtained in this way [4]. Many active substances registered as drugs (e.g., ethinyloestradiol, levonorgestrel, selegiline, pargyline, rasagiline, pralatrexate, erlotinib, ponatinib, terbinafine, tazarotene) have internal or terminal alkynyl groups [5]. This is because a triple bond, due to its unique properties, can perform many different functions, directly or indirectly influencing the biological activity of chemical compounds. This may be associated with the impact on their metabolism and their interaction with cellular proteins, and by determining the appropriate spatial structure of the molecule [6]. The phosphonate group containing a direct bond between the carbon and phosphorus atom is isosteric to the phosphate group commonly found in living organisms. The introduction of this group into the molecules of biologically active substances can lead to a significant improvement in their therapeutic properties. The P–C bond is much more resistant to breaking than the P–O bond, thanks to which the phosphonate derivatives are more stable under the conditions of enzymatic hydrolysis. Among the phosphonate derivatives used in pharmacotherapy, tenofovir disoproxil or fosfomycin can be mentioned [7,8]. The synthesis of phosphonic acid esters is of great importance in the design of new drug substances because they show better penetration through biological membranes than free phosphonic acids [9]. Introducing various new structural elements into the betulin molecule is related to the search for compounds that are characterized by better pharmacokinetic parameters while maintaining biological activity. Previously conducted studies of the 28-propynoyl derivative of betulin EB5 and its 29-diethyl phosphonate analog ECH147 against various tumor cell lines have shown promising results (Table S1; Supplementary Materials) [10,11,12,13,14,15,16]. It should be noted that the anticancer activity of betulin and its derivatives may result from their effect on mitochondria due to their hydrophobic nature and then the induction of oxidative stress [17].

A significant advantage of the potential use of betulin in cancer therapy is its low toxicity to whole living organisms [18]. Many anticancer drugs exhibit numerous side effects, such as cardiotoxicity, hepatotoxicity, and nephrotoxicity [19]. Mechanisms of drug-induced nephrotoxicity include endothelial cell dysfunction, drug accumulation in the proximal renal tubules, and increased oxidative stress in proximal and distal tubular cells [19].

For example, cisplatin causes tubular and interstitium damage as well as vascular injury. The main molecular mechanism of its action is not only the disturbance of DNA synthesis and cell cycle arrest but also the generation of reactive oxygen species (ROS) [20]. 5-fluorouracil is also suggested to be involved in the generation of free radicals; however, its influence on the oxidative status of normal cells is not fully understood [21]. Due to the proven nephrotoxicity of the currently used drugs, research on the biological activity of anti-tumor compounds should also include the study of their effects on normal cells. One of the many aspects that require clarification is the effect of these compounds on the antioxidant status of kidney cells. Such studies could shed light on the mechanisms involved in cellular defense. Therefore, the aim of our research was to determine whether betulin and its derivatives—28-propynoyl derivative of betulin EB5 and its 29-diethyl phosphonate analog ECH147—change the oxidative status of human renal proximal tubule epithelial cells (RPTECs). For this purpose, we evaluated the total antioxidant capacity of cells, the lipid peroxidation level, and the activity of antioxidant enzymes (SOD, CAT, GPX). Investigating the influence of compounds on the transcriptional activity of genes encoding antioxidant enzymes may provide additional information about the molecular mechanisms of their action. Therefore, we assessed the mRNA level of genes encoding antioxidant enzymes. Cisplatin (cis-diamminedichloroplatinum (II)-CDDP) and 5-fluorouracil (5-FU), as drugs causing oxidative stress, were used as reference substances. To date, no such studies have been carried out on betulin and its derivatives EB5 and ECH147.

## 2. Results

### 2.1. RPTECs Viability Assessment

**Figure 1:**
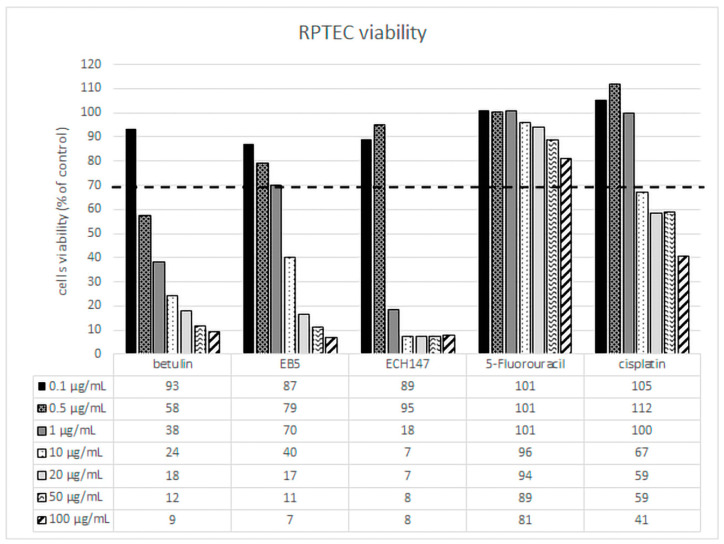
Renal proximal tubule epithelial cells viability (% of control cells) after treatment with betulin, its derivatives (EB5, ECH147), and 5-fluorouracil and cisplatin. The dashed line is the cut-off for 70% cell viability.

The reduction in cell viability below 70% proves the cytotoxic effect of the compound at a given concentration. For renal proximal tubule epithelial cells, the mildest compound was 5-fluorouracil (Figure 1). None of the tested concentrations of 5-fluorouracil resulted in a decrease in cell viability below 70%. Betulin, EB5, and ECH147 strongly affected RPTECs viability, especially at higher concentrations. EB5 at concentrations of 0.1, 0.5, and 1 μg/mL was not toxic for renal cells. Similarly, cisplatin was also safe at these concentrations; however, higher concentrations of EB5 were more toxic than cisplatin. ECH147 was safe only at concentrations of 0.1 and 0.5 μg/mL. Betulin at concentrations greater than or equal to 0.5 μg/mL caused a decrease in cell viability below 70%. This result shows that betulin and its derivatives may cause side effects in kidneys, even more severely than 5-fluorouracil and cisplatin. Thus, therapeutic concentrations should be selected with caution.

Based on the MTT assay results for further analysis, 0.1 and 0.5 μg/mL concentrations of the tested compounds were chosen.

### 2.2. Total Antioxidant Capacity of RPTECs

**Figure 2:**
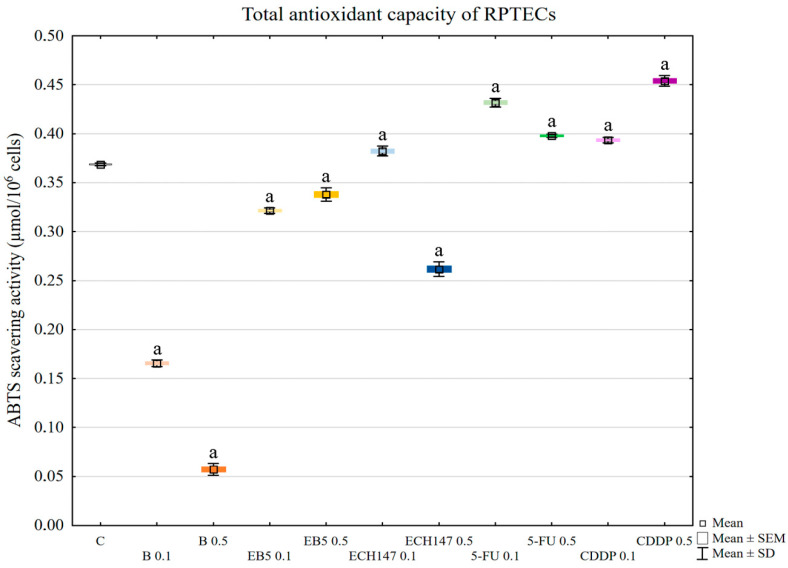
Total antioxidant capacity of renal proximal tubule epithelial cells after treatment with betulin (B), its derivatives (EB5, ECH147), 5-fluorouracil (5-FU), and cisplatin (CDDP). Data are the mean and standard deviation of the mean of three biological and three technical replicates; ^a^ *p* < 0.0005 versus control. C—control; ● B0.1—betulin at 0.1 μg/mL conc.; ● B0.5—betulin at 0.5 μg/mL conc.; ● EB50.1—EB5 at 0.1 μg/mL conc.; ● EB50.5—EB5 at 0.5 μg/mL conc.; ● ECH1470.1—ECH147 at 0.1 μg/mL conc.; ● ECH1470.5—ECH147 at 0.5 μg/mL conc.; ● 5-FU0.1—5-fluorouracil at 0.1 μg/mL conc.; ● 5-FU0.5—5-fluorouracil at 0.5 μg/mL conc.; ● CDDP0.1—cisplatin at 0.1 μg/mL conc.; ● CDDP0.5—cisplatin at 0.5 μg/mL conc.

Each of the tested compounds influenced the total antioxidant capacity (TEAC-Trolox equivalent antioxidant capacity) of RPTEC cells, compared to controls (*p* < 0.0005 in all cases) (Figure 2). Betulin strongly reduced TEAC in a concentration-dependent manner (B 0.1 μg/mL vs. B 0.5 μg/mL: *p* < 0.0002). The same effect was noted also for EB5; however, a lower concentration caused a stronger reduction compared to a higher concentration (EB5 0.1 μg/mL vs. EB5 0.5 μg/mL: *p* < 0.0002). ECH147 at a lower concentration increased TEAC (*p* = 0.0005), while a higher concentration caused a decrease in TEAC (*p* < 0.0002), compared to control cells. The difference in the antioxidant capacity of RPTECs was also statistically significant depending on ECH147 concentration (0.1 μg/mL vs. 0.5 μg/mL: *p* = 0.0002). Both 5-fluorouracil and cisplatin caused an increase of TEAC in RPTEC cells (*p* < 0.0002) compared to controls. Cisplatin at 0.1 μg/mL concentration increased TEAC compared to 0.5 μg/mL; the opposite effect was seen with 5-fluorouracil (*p* < 0.0002). This result shows that betulin and its derivatives may strongly burden the antioxidant system of renal cells.

### 2.3. Lipid Peroxidation

**Figure 3:**
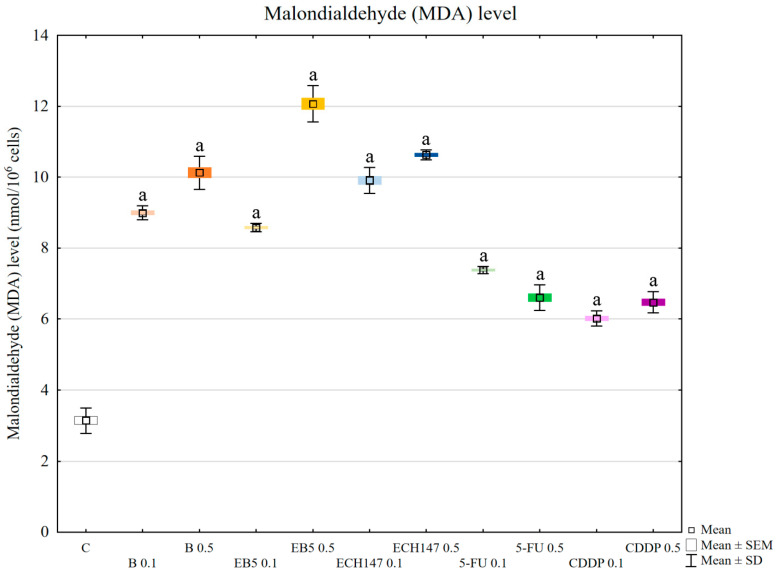
Malondialdehyde (MDA) level in renal proximal tubule epithelial cells after treatment with botulin (B), its derivatives (EB5, ECH147), 5-fluorouracil (5-FU), and cisplatin (CDDP). Data are the mean and standard deviation of the mean of three biological and three technical replicates; ^a^ *p* < 0.0005 versus control. C—control; ● B0.1—betulin at 0.1 μg/mL conc.; ● B0.5—betulin at 0.5 μg/mL conc.; ● EB50.1—EB5 at 0.1 μg/mL conc.; ● EB50.5—EB5 at 0.5 μg/mL conc.; ● ECH1470.1—ECH147 at 0.1 μg/mL conc.; ● ECH1470.5—ECH147 at 0.5 μg/mL conc.; ● 5-FU0.1—5-fluorouracil at 0.1 μg/mL conc.; ● 5-FU0.5—5-fluorouracil at 0.5 μg/mL conc.; ● CDDP0.1—cisplatin at 0.1 μg/mL conc.; ● CDDP0.5—cisplatin at 0.5 μg/mL conc.

All tested compounds increased the level of the lipid peroxidation product malondialdehyde (MDA) compared to control cells (*p* < 0.0002) (Figure 3). Interestingly, betulin and its derivatives increased the level of MDA compared to cisplatin and 5-fluorouracil (*p* < 0.0002). MDA levels were comparable for betulin and EB5 at 0.1 μg/mL concentration (*p* > 0.05). Cisplatin and 5-fluorouracil at a concentration of 0.5 μg/mL caused a comparable increase in MDA level (*p* > 0.05). These results suggest that the tested compounds may generate free radicals in a dose-dependent manner. Importantly, the effect also depends on the drug itself, which is likely due to differences in their mechanisms of action.

### 2.4. Superoxide Dismutase (SOD) Activity

**Figure 4:**
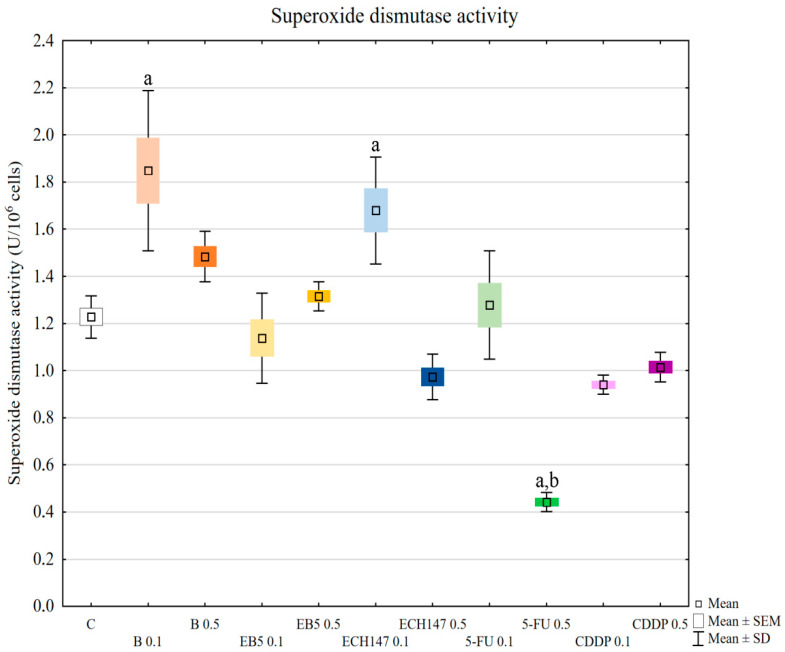
Superoxide dismutase (SOD) activity in renal proximal tubule epithelial cells after treatment with botulin (B), its derivatives (EB5, ECH147), 5-fluorouracil (5-FU), and cisplatin (CDDP). Data are the mean and standard deviation of the mean of three biological and three technical replicates; ^a^ *p* < 0.01 versus control; ^b^ *p* < 0.001 versus other compounds. C—control; ● B0.1—betulin at 0.1 μg/mL conc.; ● B0.5—betulin at 0.5 μg/mL conc.; ● EB50.1—EB5 at 0.1 μg/mL conc.; ● EB50.5—EB5 at 0.5 μg/mL conc.; ● ECH1470.1—ECH147 at 0.1 μg/mL conc.; ● ECH1470.5—ECH147 at 0.5 μg/mL conc.; ● 5-FU0.1—5-fluorouracil at 0.1 μg/mL conc.; ● 5-FU0.5—5-fluorouracil at 0.5 μg/mL conc.; ● CDDP0.1—cisplatin at 0.1 μg/mL conc.; ● CDDP0.5—cisplatin at 0.5 μg/mL conc.

The superoxide dismutase activity increased after treatment with betulin at 0.1 μg/mL (*p* = 0.0002) and ECH147 at 0.1 μg/mL concentration (*p* = 0.0008), in comparison to control cells (Figure 4). 5-fluorouracil at 0.5 μg/mL concentration caused a strong reduction in enzyme activity, in comparison to control cells and cells treated with other compounds (*p* = 0.0002). Enzyme activity in cells treated with different doses of the tested compounds (0.1 μg/mL vs. 0.5 μg/mL) was significantly different for betulin (*p* = 0.0130), ECH147 (*p* = 0.0002), and 5-fluorouracil (*p* = 0.0002).

### 2.5. Glutathione Peroxidase (GPx) Activity

**Figure 5:**
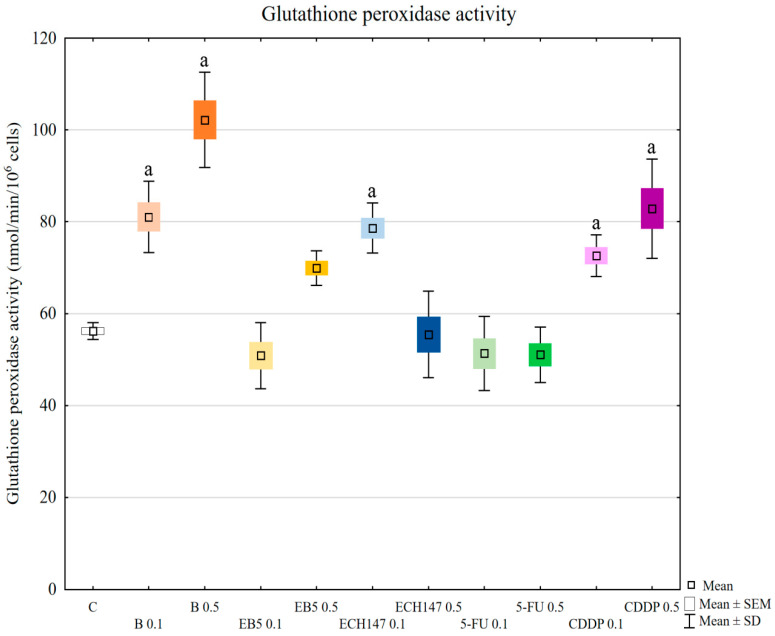
Glutathione peroxidase (GPx) activity in renal proximal tubule epithelial cells after treatment with betulin (B), its derivatives (EB5, ECH147), 5-fluorouracil (5-FU), and cisplatin (CDDP). Data are the mean and standard deviation of the mean of three biological and three technical replicates; ^a^ *p* < 0.01 versus control. C—control; ● B0.1—betulin at 0.1 μg/mL conc.; ● B0.5—betulin at 0.5 μg/mL conc.; ● EB50.1—EB5 at 0.1 μg/mL conc.; ● EB50.5—EB5 at 0.5 μg/mL conc.; ● ECH1470.1—ECH147 at 0.1 μg/mL conc.; ● ECH1470.5—ECH147 at 0.5 μg/mL conc.; ● 5-FU0.1—5-fluorouracil at 0.1 μg/mL conc.; ● 5-FU0.5—5-fluorouracil at 0.5 μg/mL conc.; ● CDDP0.1—cisplatin at 0.1 μg/mL conc.; ● CDDP0.5—cisplatin at 0.5 μg/mL conc.

In comparison to control cells, betulin and cisplatin increased glutathione peroxidase activity, regardless of concentration (*p* < 0.01) (Figure 5). ECH147 also caused an increase in enzyme activity; however, the increase was only at 0.1 μg/mL concentration (*p* = 0.0003). EB5 and 5-fluorouracil at both concentrations, and ECH147 at 0.5 μg/mL concentration, did not change GPx activity in renal cells, compared to the control. Comparing the enzyme activity in cells treated with different doses of the tested compounds (0.1 μg/mL vs. 0.5 μg/mL), statistically significant differences were observed for betulin (*p* = 0.0005), EB5 (*p* = 0.0018), and ECH147 (*p* = 0.0002).

### 2.6. Catalase (CAT) Activity

**Figure 6:**
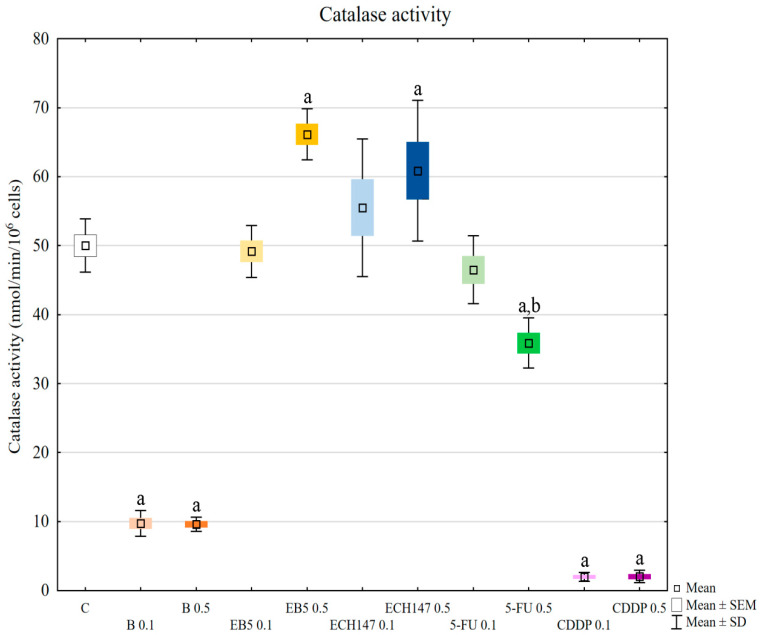
Catalase (CAT) activity in renal proximal tubule epithelial cells after treatment with betulin (B), its derivatives (EB5, ECH147), 5-fluorouracil (5-FU), and cisplatin (CDDP). Data are the mean and standard deviation of the mean of three biological and three technical replicates; ^a^ *p* < 0.001 versus control; ^b^ *p* < 0.05 versus other compounds. C—control; ● B0.1—betulin at 0.1 μg/mL conc.; ● B0.5—betulin at 0.5 μg/mL conc.; ● EB50.1—EB5 at 0.1 μg/mL conc.; ● EB50.5—EB5 at 0.5 μg/mL conc.; ● ECH1470.1—ECH147 at 0.1 μg/mL conc.; ● ECH1470.5—ECH147 at 0.5 μg/mL conc.; ● 5-FU0.1—5-fluorouracil at 0.1 μg/mL conc.; ● 5-FU0.5—5-fluorouracil at 0.5 μg/mL conc.; ● CDDP0.1—cisplatin at 0.1 μg/mL conc.; ● CDDP0.5—cisplatin at 0.5 μg/mL conc.

Betulin and cisplatin strongly reduced catalase activity, regardless of concentration, in comparison to control cells (*p* < 0.0002) (Figure 6). EB5, ECH147, and 5-fluorouracil at 0.1 μg/mL concentration did not significantly change the activity of enzymes (*p* > 0.05). At concentrations of 0.5 μg/mL, EB5 and ECH147 caused significant increases in catalase activity (*p* = 0.0002 and 0.0219, respectively), while 5-fluorouracil diminished enzyme activity (*p* = 0.0008). Statistically significant differences were also noted when comparing the enzyme activity in cells treated with different doses of the tested compounds: for EB5 0.1, μg/mL vs. 0.5 μg/mL *p* = 0.0002, and for 5-fluorouracil, 0.1 μg/mL vs. 0.5 μg/mL *p* = 0.0275.

This result showed a similar effect of betulin and cisplatin on catalase activity, resulting in its reduction. However, concerning the strong reduction of the total antioxidant capacity of cells along with the increase of the MDA level, betulin can be a major threat to maintaining a proper antioxidant defense system in renal cells. Chemical modifications of betulin (EB5 and ECH147) seem to change these properties.

### 2.7. MRNA Level of CAT, GPX3, SOD1, and SOD2 Genes

**Figure 7:**
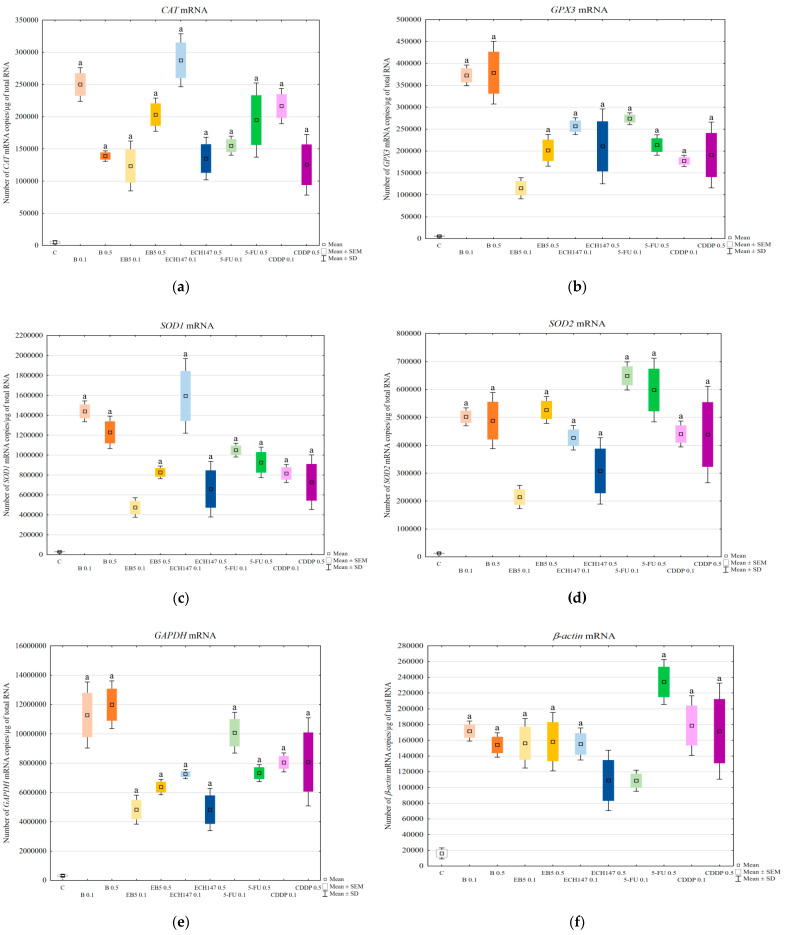
mRNA level of *CAT* (**a**), *GPX3* (**b**), *SOD1* (**c**), *SOD2* (**d**), *GAPDH* (**e**), and *β-actin* (**f**) genes in renal proximal tubule epithelial cells after treatment with betulin (B), its derivatives (EB5, ECH147), 5-fluorouracil (5-FU), and cisplatin (CDDP). Data are the mean and standard deviation of the mean of three biological and three technical replicates; ^a^ *p* < 0.05 versus control. C—control; ● B0.1—betulin at 0.1 μg/mL conc.; ● B0.5—betulin at 0.5 μg/mL conc.; ● EB50.1—EB5 at 0.1 μg/mL conc.; ● EB50.5—EB5 at 0.5 μg/mL conc.; ● ECH1470.1—ECH147 at 0.1 μg/mL conc.; ● ECH1470.5—ECH147 at 0.5 μg/mL conc.; ● 5-FU0.1—5-fluorouracil at 0.1 μg/mL conc.; ● 5-FU0.5—5-fluorouracil at 0.5 μg/mL conc.; ● CDDP0.1—cisplatin at 0.1 μg/mL conc.; ● CDDP0.5—cisplatin at 0.5 μg/mL conc.

All tested compounds caused an increase in the transcriptional activity of genes encoding antioxidant enzymes and endogenous controls (Figure 7). Statistically significant differences were noted comparing the mRNA level in cells treated with different doses of the tested compounds (0.1 μg/mL vs. 0.5 μg/mL): for *CAT* gene betulin (*p* = 0.0258) and ECH147 (*p* = 0.0004), for *SOD1* gene ECH147 (*p* = 0.0002), for *SOD2* gene EB5 (*p* = 0.0080), and for *β-actin* gene 5-fluorouracil (*p* = 0.0028).

### 2.8. Correlation Coefficient between Tested Parameters

In cells treated with EB5 at 0.5 μg/mL concentration, a positive correlation in catalase and glutathione peroxidase activity was found (r = 0.8118, *p* = 0.0498).

A positive correlation in superoxide dismutase and glutathione peroxidase activity was found in cells treated with betulin at 0.1 μg/mL concentration (r = 0.8771, *p* = 0.0217), EB5 at 0.1 μg/mL concentration (r = 0.8181, *p* = 0.0466), and cisplatin at 0.5 μg/mL concentration (r = 0.8674, *p* = 0.0252).

## 3. Discussion

Currently available anticancer drugs are not always effective, which is due, among other things, to the genetic changes that characterize tumor tissue. Additionally, many drugs with anti-tumor activity, despite being effective against tumor cells, exhibit several serious side effects. One such effect is nephrotoxicity, which results from endothelial cell dysfunction and increased oxidative stress in proximal and distal tubular cells [19]. Therefore, compounds with high anti-tumor activity and, at the same time, low cytotoxicity against normal cells are constantly sought after. One of the currently used strategies is the search for such drugs among compounds of natural origin. Additionally, the introduction of chemical modifications makes it possible to improve properties, such as anticancer activity, stability, and pharmacokinetic parameters. One of the most promising compounds of natural origin is betulin [2,22]. Its availability and the ease of obtaining it from birch bark encourage the search for new therapeutic solutions [1]. Betulin modifications, such as the 28-propyne derivative EB5 and its 29-diethyl phosphonate analog ECH147, showed promising results in anti-tumor activity studies [10,11,12,13,14,15,16]. Despite the promising results of research on the biological activity of betulin and its derivatives, these compounds, so far, have not been used in pharmacotherapy due to their low bioavailability resulting from limited solubility in the water environment. The conversion of the hydroxyl groups present in the betulin molecule into other functional groups may result in a reduction of the hydrophobicity.

A commonly used method of determining the bioavailability of new drug substances is predicting their properties by determining theoretical values of pharmacokinetic parameters such as lipophilicity. For betulin and its derivatives EB5 and ECH147, the value of the partition coefficient (logP) was calculated using the internet database VCCLAB [23,24]. The logP values determined with the use of the XLOGP3 algorithm are 8.28, 9.34, and 8.83 for betulin, EB5, and ECH147, respectively. The lipophilicity of the 28-propynoylated derivatives determined in this way is quite high and comparable to the lipophilicity of the parental betulin.

Studies on the mechanism of the anticancer action of triterpene derivatives indicate the possibility of induction of apoptosis through the mitochondrial (intrinsic) pathway. These compounds contribute to the reduction of the mitochondrial outer membrane potential (MOMP) and the production of reactive oxygen species (ROS). The ROS produced by the mitochondria may cause the release of pro-apoptotic proteins that trigger apoptosis or necrosis [25].

Designing selective anticancer compounds targeting the mitochondria is related to the development of molecules that can increase the permeability of the MOMP. The possibility of obtaining betulin derivatives targeted at mitochondria is related to the introduction of a triphenylphosphonium moiety into its structure or transforming it into a conjugate with rhodamine B or cation F16 [26]. The lipophilic cation F16 (E-4-(1H-indol-3-ylvinyl)-N-methylpyridinium iodide) is a promising molecule that targets mitochondria and shows free-form anticancer activity. The combinations of the F16 cation with the molecules of betulin or betulinic acid exhibiting anticancer activity show a significantly enhanced anti-tumor effect. These conjugates target the mitochondria by inducing an overproduction of ROS and a permeabilization of organelle membranes [27].

However, the effect of betulin and its derivatives on kidney cells has not yet been studied, which is extremely important due to its potential nephrotoxicity.

Studies on betulin toxicity to normal cells are sparse, and the results are often inconclusive. Moreover, they have, so far, been carried out mainly on cell lines derived from animals. Studies conducted by Zehra et al. on normal mouse fibroblasts (NIH-3T3) showed low toxicity of betulin [28]. On the other hand, studies by Małaczewska et al. have shown that betulin is highly toxic to NIH-3T3, and these cells were more sensitive to betulin than fish fibroblasts BF-2 [29]. This study showed the different sensitivity of the cells, depending on the species origin. It is vital to choose a proper in vitro model for drug toxicity studies [30]. The sensitivity of cells may depend on their type and tissue origin. Thus, the cell line should be selected to best reflect the properties of the target tissue.

In our study, we used a model of human renal proximal tubule epithelial cells to evaluate the nephrotoxicity of betulin and its derivatives. These cells are isolated from normal human kidney tissue and are not genetically modified in any way. The use of such a research model allows the assessment of the cytotoxicity of compounds that best reflect their effects on normal human cells. We used two reference drugs: cisplatin and 5-fluorouracil. Cisplatin is a drug with a proven nephrotoxic effect resulting from proximal tubular dysfunction [19], but the effect of 5-fluorouracil on kidney cells has not been clearly defined. In studies of the protective effects of compounds of natural origin in animal models, 5-fluorouracil has been used as a drug to induce kidney damage [21]. However, despite its numerous side effects, 5-FU does not cause nephrotoxicity. Clinical reports indicate that nephrotoxicity occurs with 5-FU therapy in combination with other drugs [31]. Therefore, in our studies, we assumed that 5-FU would slightly affect the viability of the human renal tubular proximal epithelial cells. The results confirmed that 5-FU is not toxic to RPTECs in the concentrations and incubation times we tested. RPTECs showed greater sensitivity to betulin and its derivatives than 5-fluorouracil. Cisplatin was toxic at higher concentrations. It should be emphasized that the chemical modifications introduced to the betulin molecule (EB5 and ECH147) resulted in a slightly better RPTEC tolerance to the concentrations used. Based on these results, two compound concentrations of 0.1 µg/mL and 0.5 µg/mL were selected for further studies.

In the next stage, the total antioxidant capacity of the RPTECs was assessed. Betulin strongly lowered the antioxidant capacity of kidney cells. EB5 also lowered the antioxidant potential, but not to the same extent as betulin, while ECH147 lowered the potential only at the higher concentration used (0.5 µg/mL). Interestingly, cisplatin and 5-fluorouracil increased the antioxidant potential of the RPTECs. This result was particularly surprising in the case of cisplatin, the mechanism of action of which is based, inter alia, on the induction of oxidative stress [20]. Perhaps crucial in our analysis was the 24-h incubation time, which was insufficient to induce strong oxidative stress in tubular cells. Cisplatin-dependent generation of ROS results from mitochondrial dysfunction, DNA damage, and induction of an inflammatory response [20]. Moreover, in tubular cells, cisplatin undergoes a series of reactions, resulting in the generation of toxic metabolites. It is likely that the oxidative stress in RPTEC cells after 24 h of incubation with cisplatin gradually increased, and in the initial stage, induced the antioxidant response. Low concentrations of ROS are known to activate signaling pathways responsible for the antioxidant defense of cells [32]. Moreover, intracellular components may interact with cisplatin, resulting in its inactivation [20]. Regardless of the mechanism responsible for the response of cells to cisplatin, it should be emphasized that, in our study, betulin significantly decreased the total antioxidant capacity of tubular cells, which suggests that this compound strongly burdens the antioxidant system of tubular cells during the first 24 h.

To evaluate the effect of oxidative stress induction on cellular molecules, malondialdehyde (MDA) levels were assessed. All tested compounds increased the level of MDA in RPTEC cells; however, betulin and its derivatives had a greater effect than the reference drugs. This result confirmed that oxidative stress caused by betulin and its derivatives during 24 h of incubation is stronger than that caused by cisplatin and 5-FU. Malondialdehyde is a product of lipid peroxidation, and its level is used as a marker of kidney damage [33]. Moreover, MDA is highly reactive toward nucleophiles, resulting in the formation of MDA adducts with important cellular proteins [34]. These, in turn, may influence intracellular signal transduction. Such an interaction was found for protein kinase C (PKC), which is involved in signal transduction, resulting in the modulation of vital processes, such as proliferation, inflammation, and cytoskeletal organization [34].

Another important stage of our research was the assessment of the activity of superoxide dismutase (SOD), glutathione peroxidase (GPx), and catalase (CAT). They are the main antioxidant enzymes in cells [35]. The first line of antioxidant defense is the dismutation of a superoxide anion radical to oxygen and hydrogen peroxide catalyzed by SOD [33]. There are three isoforms of superoxide dismutases in human cells: cytoplasmic SOD1, mitochondrial SOD2, and extracellular SOD3; however, each catalyzes the same reaction. GPx, using glutathione, reduces hydrogen peroxide and hydroperoxides in water and alcohol [35]. Thus, GPx eliminates hydrogen peroxide and lipid peroxides. It is also found mainly in cytosol, mitochondria, and in nuclei. CAT is a peroxisomal enzyme that converts hydrogen peroxide to water and oxygen [35]. One of the primary sources of ROS in cells is the mitochondrial respiratory chain. Dubinin et al. [17,27] showed that betulin and its derivatives affected mitochondrial membranes and inhibited mitochondrial respiratory chain complexes, which is accompanied by an increased production of ROS. The authors also note that this may be the cause of the cytotoxicity of these agents.

In our research, the assessment of SOD, GPx, and CAT activity revealed different reactions of RPTECs to the compounds used. Betulin and cisplatin caused an increase in glutathione peroxidase activity. Due to its activity leading to hydrogen peroxide and lipid peroxide elimination, this result may justify the rapid increase of these molecules in cells treated with betulin and cisplatin. Adversely, betulin and cisplatin strongly diminished catalase activity compared to the control cells and cells treated with other compounds. Catalase depletion results in ROS accumulation in mitochondria and functional impairment, leading to kidney damage [33]. Moreover, this enzyme prevents lipid peroxidation. Therefore, the reduction in catalase activity by betulin, comparable to that observed for cisplatin, supports the nephrotoxic potential of betulin. This mechanism probably involves the functional impairment of mitochondria. Importantly, at higher concentrations, betulin derivatives EB5 and ECH147 increased the activity of catalase, which gives hope that these compounds will not cause serious consequences for kidney cells in vivo. SOD activity was increased in cells treated with betulin and ECH147 at lower concentrations, compared to the control and cells treated with other compounds. The inhibition of SOD activity results in an increased generation of ROS in kidney cells [33]. However, clinical studies concerning SOD levels in chronic kidney disease patients have provided contradictory results [36]. Since SOD is the first enzyme to respond to oxidative stress, an increase in its activity in cells treated with betulin and ECH147 in the first 24 h may be the result of a strong cell reaction to unfavorable conditions. However, considering the other parameters determined by us, ECH147 probably does not cause such strong changes in the antioxidant system as betulin. These results confirm that a 24-h exposure to betulin significantly affects the efficiency of antioxidant systems of human renal proximal tubule epithelial cells. Moreover, each of the tested compounds revealed different profiles of changes in the assessed parameters.

Since all tested compounds increased the level of MDA, which can, in turn, modulate intracellular signaling, in the next step, we checked whether changes in the transcription level of genes encoding antioxidant enzymes occurred. We determined the mRNA levels of the following genes: *CAT*, *SOD1*, *SOD2,* and *GPX3*. The level of mRNAs of genes encoding antioxidant enzymes was increased in all treated cells, regardless of the compound used. Betulin strongly activated *GPX3* gene transcription. We selected *GPX3* for analysis because of its strong expression in the kidneys [37,38]. However, its function in terms of drug-induced oxidative stress has not been fully elucidated. Shirota et al. showed that H_2_O_2_ induces GPx3 expression in mice renal tubular cells, both at the mRNA and protein levels [38]. Thus, the strong expression of the *GPX3* gene at the mRNA level in RPTECs treated with betulin confirmed the induction of oxidative stress. The expression of *SOD1*, *SOD2,* and *CAT* genes was also impaired by the tested compounds, suggesting the activation of defense mechanisms. The intensification of the expression of genes encoding antioxidant enzymes is aimed at guaranteeing the appropriate number of molecules at the protein level, which, in turn, should result in the reduction of oxidative stress. The expression profiles of the studied genes were different for individual compounds and depended on their doses. Thus, the result of prolonged exposure of RPTECs needs further research. We also detected the influence of the tested compounds on the transcriptional activity of the endogenous control genes *GAPDH* and *β-actin*. Like the genes encoding antioxidant enzymes, the transcriptional activity of the endogenous control genes was increased by all tested compounds. We have previously reported that the mRNA levels of *GAPDH* and *β-actin* genes changed in RPTECs depending on AmB modification treatment [39]. Therefore, 18S rRNA was used as an endogenous control [39]. However, in our present work, we were unable to use this parameter for relative quantification because, as in the case of *GAPDH* and *β-actin* genes, its expression changed under the influence of the compounds used (data not shown). Unfortunately, in the literature, we have not found data indicating which reference gene would meet all the required criteria [40] and would remain unchanged under the influence of all compounds tested by us. This confirmed the observation made by Pfaffl, that the selection of an appropriate reference gene is a crucial problem in the relative quantification method [41].

Therefore, we have used the absolute quantification method and the results were calculated based on a standard curve, as was described previously by Strzalka-Mrozik et al. [42]. This method is not ideal, but each method used to determine changes in the gene expression has its own advantages and disadvantages [43].

It is also worth emphasizing that, in our previous work [39], we used both methods of gene expression quantification (absolute and relative) and proved that both methods confirmed the changes in the expression of the analyzed genes.

## 4. Materials and Methods

### 4.1. EB5 and ECH147 Synthesis

The 28-propynoyl-substituted derivatives of betulin EB5 and ECH147 were synthesized in the Department of Organic Chemistry, Faculty of Pharmaceutical Sciences in Sosnowiec (SUM), as earlier described in the literature [10,44]. Betulin 1 was used as substrate to obtain the 28-propynoylbetulin EB5 and the 29-diethoxyphosphoryl-28-propynoylbetulin ECH147 (Scheme S1, Supplementary Materials). To obtain derivative ECH147, betulin 1 was first converted into the diethylphosphonate derivative. The several-step synthesis was started by the reaction of betulin 1 with excess acetic anhydride, which resulted in the formation of 3,28-diacetylbetulin A, which was then reacted with triethyl phosphite to obtain 30-diethoxyphosphoryl-3,28-diacetylbetulin B. Hydrolysis of product B with KOH in boiling ethanol led to deblocking hydroxyl groups at positions 3 and 28, but also caused the allyl–vinyl isomerization of the isopropenyl group. The (*E)*-isomer of 29-diethoxyphosphorylbetulin C, which was formed in greater quantity, was selected for the last stage of the synthesis. Esterification reaction of betulin 1 or 29-diethoxyphosphorylbetulin C with propynoic acid provided the corresponding 28-propynoyl-substituted derivatives of betulin (EB5 and ECH147, Scheme S1). Crude reaction products were purified by column chromatography. The structures of the synthesized compounds were determined based on their ^1^H-^13^C- and ^31^P-NMR (Bruker AVANCE III HD 600, Billerica, MA, USA, deuterated chloroform), IR (IRAffinity-1 FTIR spectrometer; Shimadzu Corporation, Kyoto, Japan, KBr pellet), and MS spectra (Bruker Impact II, Billerica, MA, USA). MThe melting point, R_f_ parameters, and spectroscopic (^1^H, ^13^C, and ^31^P NMR) data for the tested compounds were consistent with the literature information [10,44]. (The most important data characterizing compounds EB5 and ECH147 are presented in Table S2, Supplementary Materials).

### 4.2. Cell Culture Conditions and RPTECs Viability Assessment

Normal human RPTECs (CC-2553, Lonza, Basel, Switzerland) were routinely maintained at 37 °C in a 5% CO2 incubator (Direct Heat CO_2_; Thermo Scientific, Waltham, MA, USA) with the use of a REGM Bullet Kit (renal epithelial basal medium (REBM), supplements, and growth factors (SingleQuots) CC-3190, Lonza). The MTT (3-[4,5-dimethylthiazol-2-yl]-2,5-diphenyltetrazolium bromide) assay (Sigma–Aldrich, St Louis, MO, USA) was used to determine the influence of betulin, EB5, ECH147, cisplatin, and 5-fluorouracil on RPTECs viability. Cells were treated with tested compounds for 24 h at concentrations: 0.1, 0.5, 1, 10, 20, 50, and 100 μg/mL. Controls were left untreated. MTT (0.25 mg/mL) was added to the medium for 3 h (37 °C). After washing with PBS, cells were lysed in 100 μL of dimethyl sulfoxide (Sigma-Aldrich). Absorbance was measured at the wavelength of 540 nm with the use of microplate reader Wallac 1420 VICTOR (PerkinElmer, Waltham, MA, USA). Based on the MTT test results, two concentrations of compounds were determined for further studies: 0.1 and 0.5 μg/mL. These concentrations influenced the viability of the cells, but at the same time allowed for further research. RPTEC cells were treated with the tested compound at concentrations 0.1 and 0.5 μg/mL for 24 h. Untreated cells served as negative controls. Each variant of the experiment was performed in three biological replications.

For the assessment of the antioxidant capacity of cells, lipid peroxidation level, and activity of antioxidant enzymes, cells were suspended in lysis buffer (containing 4 mg of SIGMAFAST™ Protease Inhibitor Cocktail and 10 µL Phosphatase Inhibitor Cocktail 3, in 1 mL of phosphate-buffered saline; Sigma–Aldrich). The cell suspensions were placed in liquid nitrogen for 30 min and then stored at −80 °C until further analysis. For the assessment of the mRNA level of genes encoding antioxidant enzymes cells were lysed with the use of TRIzol (Invitrogen Life Technologies, Carlsbad, CA, USA) and then stored at −80 °C until further analysis.

### 4.3. Total Antioxidant Capacity of RPTECs

The total antioxidant status was measured using the ABTS + radical cation. The technique for the generation of ABTS + involves the direct production of the blue/green ABTS + chromophore through a reaction between ABTS (Sigma–Aldrich) and potassium persulfate (Sigma–Aldrich). The reduction of the pre-formed radical cation to ABTS depends on the antioxidant activity of cells. The extent of decolorization, as a percentage of the inhibition of the ABTS + radical cation, is determined as a function of concentration and time, and is calculated relative to the reactivity of Trolox (TEAC—Trolox equivalent antioxidant capacity). A decrease in the absorbance at 734 nm was measured [45].

### 4.4. Lipid Peroxidation (MDA Assay)

The lipid peroxidation (malondialdehyde, MDA) level was measured using Malondialdehyde Assay Kit (Aoxre Bio-Sciences, Burlingame, CA, USA), according to the manufacturer’s instructions. In this method, chromogenic reagent N-methyl-2-phenylindole (NPMI) reacts with MDA at 45 °C, forming stable carbocyanine dye. Absorbance was measured at 586 nm.

### 4.5. CAT, SOD and GPx Activity Assessment

Superoxide dismutase (SOD) activity (total SOD activity: cytosolic and mitochondrial) was determined using a Cayman Chemical’s Superoxide Dismutase Assay Kit (Cayman Chemical, Ann Arbor, MI, USA), according to the manufacturer’s protocol. Xanthine and hypoxanthine generate superoxide radicals which, when bound with a tetrazolium salt, transform it into red formazan. One unit of SOD activity is defined as the amount of the enzyme that is needed to convert 50% of the superoxide radicals. Previously prepared cell lysates were used as the test material and centrifuged after thawing, and the collected supernatant was used for further studies. The SOD activity was determined spectrophotometrically at 440–460 nm.

Glutathione peroxidase (GPx) activity was determined using a Cayman Chemical’s Glutathione Peroxidase Assay Kit (Cayman Chemical), according to the manufacturer’s protocol. The GPx activity was indirectly measured using a coupled reaction with glutathione reductase (GR (glutathione reductase). GPx catalyzes the reduction reaction of cumene hydroperoxide and, as a result, an oxidized form of glutathione is formed, which was then reduced in the presence of GR and NADPH oxidation to NADP^+^ (accompanied by a decrease in absorbance). The decrease in absorbance is directly proportional to the GPx activity in the sample. Activity was evaluated based on the spectrophotometric measurement at 340 nm.

Catalase (CAT) activity was measured using a Catalase Assay Kit (Cayman Chemical). The method is based on the reaction of CAT with methanol in the presence of an optimal concentration of H2O2. The formaldehyde that was produced was measured spectrophotometrically using 4-amino-3-hydrazino-5-mercapto-1,2,4-triazole as the chromogen. Purpald specifically forms a bicyclic heterocycle with aldehydes, which, upon oxidation, changes from colorless to purple. Colorimetric measurement was performed at 540 nm.

### 4.6. Assessment of mRNA Level of CAT, SOD1, SOD2, and GPX3 Genes

Cells lysed with TRIzol (Sigma–Aldrich, Saint Louis, MO, USA) were subjected to total RNA extraction, according to the manufacturer’s instructions. Total RNA concentration was determined using MaestroNano MN-913 (MaestroGen Inc., Las Vegas, NV, USA). The quality of RNA extracts was assessed based on agarose gel electrophoresis. The mRNA level of the studied genes was evaluated with the use of the real-time RT-qPCR method, using the GoTaq^®^ 1-Step RT-qPCR System kit (Promega, Madison, WI, USA) and specific primers (Table 1). The thermal conditions of the reaction were as follows: reverse transcription, 37 °C—15 min; activation, −95 °C—10 min, 40 cycles: denaturation, 95 °C—10 s; annealing, −60 °C—30 s; extension, 72 °C—30 s. The mRNA copy number was determined based on a standard curve method described previously by Strzalka-Mrozik et al. [42].

The RT-qPCR reaction was performed using LightCycler^®^ 480 System apparatus (Roche, Basel, Switzerland). All samples were tested in triplicate. *β-actin* and *GAPDH* mRNA expression assessment was used as positive control of amplification. The assessment of the specificity of the reaction was made by analyzing the melting point of the PCR products and electrophoresis in 2% agarose gel (Figure S1. Supplementary Materials).

### 4.7. Statistical Analysis

Statistical analysis was performed using Statistica 13.3 software (TIBCO Software Inc., Palo Alto, CA, USA). Values were expressed as the means and standard deviation (SD), the level of significance was set at *p* < *0*.05. All the biochemical parameters were recalculated for 10^6^ cells. The number of mRNA copies of each gene was recalculated per µg of total RNA. Differences among the groups were evaluated using one-way ANOVA test and Tukey’s post hoc test.

## 5. Conclusions

Our study showed that betulin and its derivatives affect the viability of human renal proximal tubule epithelial cells by influencing the antioxidant systems.

Betulin showed an effect similar to cisplatin, indicating its nephrotoxic potential. Betulin derivatives EB5 and ECH147 showed different impacts on the antioxidant system. This gives hope that these compounds will not cause severe consequences for the kidneys in vivo. Moreover, the presence of a free hydroxyl group in C3 position makes it possible to carry out further modifications consisting in the introduction of specific groups targeted at mitochondria, which may improve the bioavailability of the tested derivatives.

It should be emphasized, however, that the effect observed for the cell line during the in vitro study would not be the same for organ reactions in vivo. Thus, further research is necessary.

## Figures and Tables

**Table 1:** Primer sequences for amplification of *CAT*, *SOD1*, *SOD2*, and *GPX3* mRNAs.

Gene	Primer Sequences	Length of PCR Product (bp)
*SOD1*	F: 5′-TTGGGCAATGTGACTGCTGACAAA-3′ R: 5′-GGGCGATCCCAATTACACCACAA-3′	208
*SOD2*	F: 5′-CTGGACAAACCTCAGCCCTA-3′ R: 5′-CTGATTTGGACAAGCAGCAA-3′	199
*CAT*	F: 5′-AGAGAAATCCTCAGACACATC-3′ R: 5′-CAGCTTGAAAGTATGTGATCC-3′	161
*GPX3*	F: 5′-GCAACCAATTTGGAAAACAG-3′ R: 5′-CTCAAAGAGCTGGAAATTAGG-3′	107
*β-actin*	F: 5′-TCCGAGAGAAAACAGCCTTTT-3′ R: 5′-TCACCCACATGTGCCCATCTACGA-3′	295
*GAPDH*	F: 5′-GAAGGTGAAGGTCGGAGTC-3′ R: 5′-GAAGATGGTGATGGGATTC-3′	226

## Data Availability

Not applicable.

## Supplementary Materials

The following supporting information can be downloaded at: https://www.mdpi.com/article/10.3390/ijms23052524/s1.
