# Supplementary material for: The Influence of Betulin and Its Derivatives EB5 and ECH147 on the Antioxidant Status of Human Renal Proximal Tubule Epithelial Cells
Source: Int J Mol Sci. 2022 Feb 25;23(5):2524. doi: 10.3390/ijms23052524 (PMC8910190; doi:10.3390/ijms23052524)
Supplement: Supplementary file 1 [file ijms-23-02524-s001.zip › ijms-1575446-supplementary.pdf]

Supplementary materials

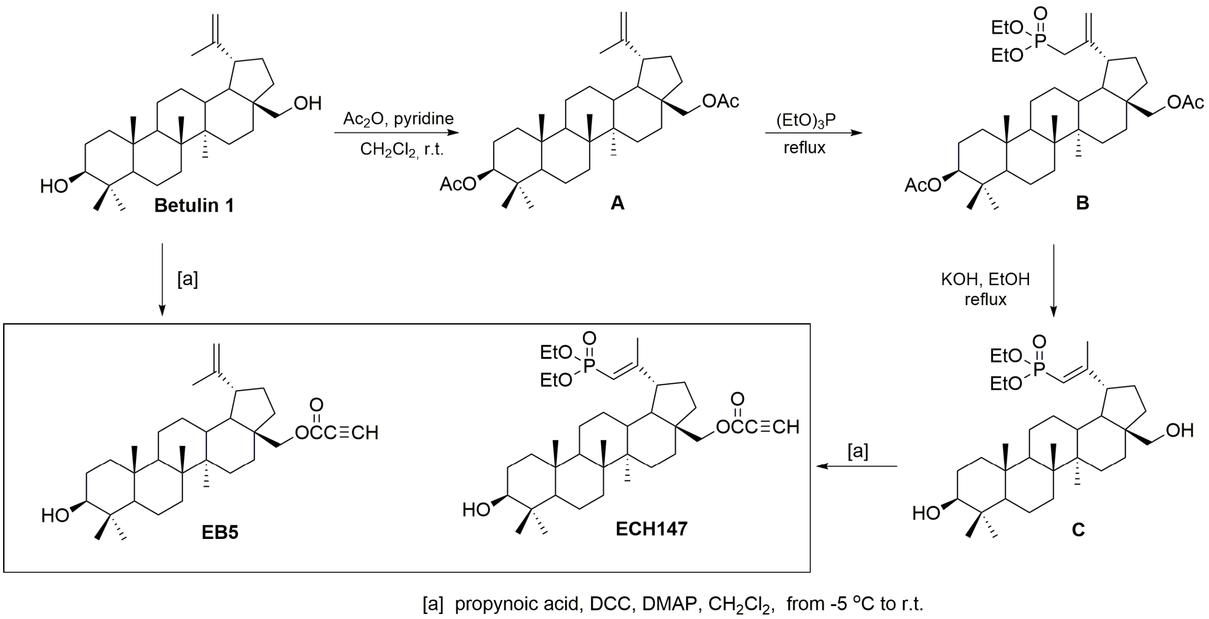

Scheme S1. Synthesis of 28-propynoyl-substituted derivatives of betulin EB5 -and EB147.

Table S1. Cytotoxic activity tested derivatives of betulin against various cancer cell lines.

Compound	Cell line	IC_{50}	[References]
**EB5**	breast cancer: T47D<br>leukemia: CCRF/CEM<br>colorectal adenocarcinoma: SW707<br>murine leukemia: P388	(18.4 μM)<br>(0.04 μM)<br>(30.2 μM)<br>(0.81 μM)	[10]
	leukemia: HL-60	(0.61 μM)	[11]
	breast cancer: MCF-7<br>glioblastoma: SNB-19<br>melanoma: Colo-829<br>melanoma: C-32	(102.1 μM)<br>(4.2 μM)<br>(1.7 μM)<br>(16.7 μM)	[12]
	breast cancer: SK-BR-3	(3.83 μM)	[13]
	glioblastoma: T98G<br>rat glioma: C6	(16.23 μM)<br>(9.11 μM)	[14]
	neuroblastoma: SK-N-AS<br>rhabdomyosarcoma: TE671	(0.62 μM)<br>(1.34 μM)	[15]
**ECH147**	breast cancer: T47D<br>glioblastoma: SNB-19<br>melanoma: C-32	(0.70 μM)<br>(0.43 μM)<br>(0.60 μM)	[16]

Table S2. Physicochemical and characteristic spectral data for compound EB5 and ECH147.

**Characteristics of compounds**	**EB5**	**ECH147**
Melting point	133–135°C	190-191°C
R_f	0.46 (chloroform/ethanol, 20:1, v/v)	0.52 (dichloromethane/ethanol, 15:1, v/v)
^{1}H-NMR (600 MHz) δ_H [ppm]	1.69 (3H, H-30); 2.45 (m, 1H, H-19); 2.91 (s, 1H, C≡C*H*); 3.21 (m, 1H, H-3); 4.01 (d, *J* = 10.8 Hz, 1H, H-28); 4.41 (d, *J* = 10.8 Hz, 1H, H-28); 4.71 (s, 1H, H-29); 4.61 (s, 1H, H-29)	1.33 (m, 6H, 2x OCH_2CH_3); 1.06–2.01 2.07 (d, *J* = 3Hz, 3H, H-30); 2.47 (m, 1H, H-19); 2.93 (s, 1H, C≡C*H*); 3.20 (m, 1H, H-3); 3.97 (d, *J* = 10.8 Hz, 1H, H-28); 4.05 (m, 4H, 2xOCH_2CH_3) 4.39 (d, *J* = 10.8 Hz, 1H, H-28); 5.43 (d, 1H, $^2J_{PH}$ = 18 Hz, H-29).
^{13}C-NMR (150 MHz) δ_C [ppm]	21.8 (C-30); 47.7 (C-19); 64.9 (C-28); 74.7 (C≡C); 74.8 (C≡C); 78.9 (C-3); 110.0 (C-29); 149.9 (C-20); 153.3 (O-C=O)	16.0 (OCH_2CH_3); 16.1 (OCH_2CH_3); 17.5 (d, $^3J_{CP}$ = 7.8 Hz, C-30); 51,4 (C-19); 61.1 (OCH_2CH_3); 61.2 (OCH_2CH_3); 64.6 (C-28); 74.6 (C≡C); 74.9 (C≡C); 78.9 (C-3); 111.9 (d, $^1J_{CP}$ = 183 Hz, C-29); 153.2 (O-C=O); 166.8 (C-20)
^{31}P-NMR (243 MHz) δ_P [ppm]	-	18.25

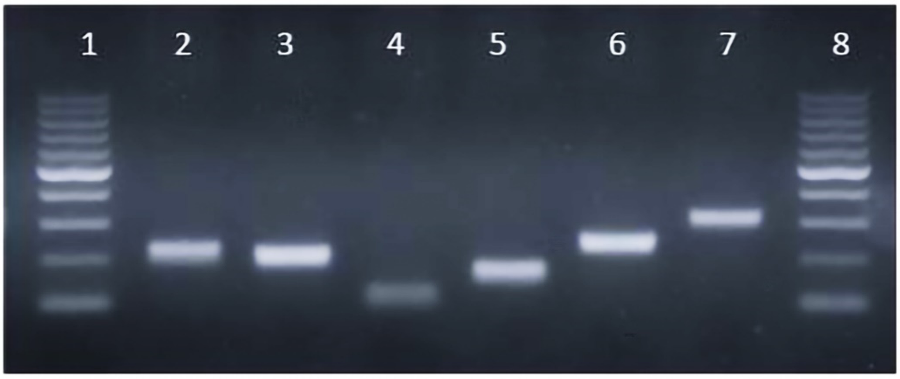

Figure S1. The result of the electrophoretic separation of RT-qPCR products. Lane 1 and 8 – product size marker (Perfect™ 100-1000 bp DNA Ladder, EURx, Polska), lane 2: *SOD1* (208 bp), lane 3: *SOD2,* (199bp), lane 4: *GPX3* (107bp), lane 5: *CAT* (161bp), lane 6: *GAPDH* (226bp), lane 7: *β-Actin* (295bp)
